# Three’s Company: A Rare Case of a Myocardial Bridge With Concomitant SCAD and Takotsubo Cardiomyopathy∗

**DOI:** 10.1016/j.jaccas.2020.12.025

**Published:** 2021-02-17

**Authors:** Eugenia Gianos, Aeshita Dwivedi

**Affiliations:** a Donald and Barbara Zucker School of Medicine at Hofstra/Northwell, Northwell Health, Hempstead, New York, USA; b Division of Cardiology, Lenox Hill Hospital, Northwell Health, New York, New York, USA

**Keywords:** myocardial bridge, spontaneous coronary artery dissection, Takotsubo’s cardiomyopathy

**Figure:**
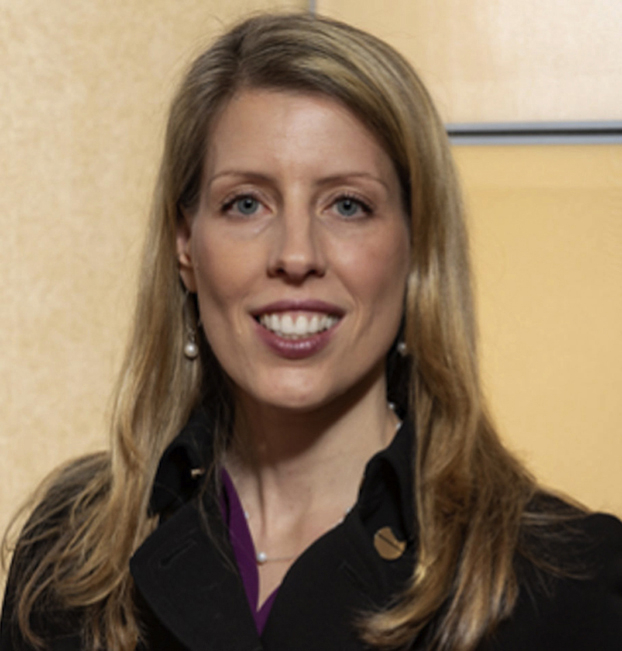

In this issue of *JACC: Case Reports*, Kegai et al. (1) present a highly unusual case of spontaneous coronary artery dissection (SCAD), Takotsubo cardiomyopathy (TC), and myocardial bridge simultaneously noted in a single patient. To our knowledge, this is a “first to be reported” finding and very aptly combines multimodality imaging, including angiography, ventriculography, and intravascular ultrasound to reach a unique diagnosis. The recognition of these findings in a single patient opens the potential for overlapping mechanisms that may allow for better understanding of the individual pathologies. This also makes us more attuned to look for this overlap in future patients where our understanding of the pathology remains limited.

In reviewing any case, it is usually most advantageous to search for a single unifying diagnosis; however, in cases where multiple individual syndromes may be evident, finding an explanation for the simultaneous presentation can be of use. Overlap of mechanisms leading concomitantly to SCAD and TC have been described in published reports (2,3). Both conditions are precipitated by emotional or physical stress and typically tend to heal over time. Kegai et al. (1) discuss the overlap in precipitating factors and how shearing forces at the hinge point of wall motion abnormalities in TC (exacerbated by vasospasm) may predispose to SCAD (4). Moreover, the presence of altered flow mechanics in a myocardial bridge have also been postulated to predispose to SCAD (5,6). Therefore, it is not inconceivable that the 3 conditions overlapped and sequentially precipitated the clinical presentation in this patient.

In review of the workup for this case, several imaging modalities proved to be most valuable. Angiography was indeed the appropriate modality in the setting of acute chest pain coupled with ST-segment elevations on electrocardiography, even if SCAD or TC was suspected. Obstructive coronary artery disease must be ruled out before the aforementioned diagnoses can be established. Kegai et al. (1) elected to perform balloon angioplasty in the setting of ongoing chest pain, which is reasonable. In most cases of SCAD, however, it is preferable not to intervene because the intervention may cause more harm (7). Although IVUS was useful in confirming the diagnosis of SCAD and guided clinical management by noting concomitant atherosclerosis, it is not without risks (8), and SCAD was clearly noted on angiography. As a matter of fact, between the coronary imaging and ventriculography, all 3 diagnoses were fairly well illustrated through left heart catheterization alone.

Repeat angiography at day 6 with acetylcholine challenge showed healing of the dissection with evidence of vasospasm, which also guided clinical management. The published reports to date for SCAD show that repeat angiography is not indicated, however, and may potentially cause harm in further propagating a dissection with the use of guidewires, balloons, or stents (7,9). Similarly, acetylcholine should likely be avoided in a recent dissection where the mechanism was believed to involve vasospasm. Although coronary computed tomography angiography has a limited role in the diagnosis of SCAD (10), because dissections in the smaller arteries may be missed (because of limited spatial resolution), it is helpful to assess for the resolution of coronary dissections if clinically indicated (11). The diagnosis of TC was very nicely illustrated in the systolic phase of the ventriculogram, showing apical ballooning with hyperdynamic basal segments. An echocardiogram was also of high yield and low risk, additionally revealing left ventricular outflow tract obstruction, commonly seen in the setting of TC. Echocardiography is also essential in such cases to evaluate for any complications of TC other than left ventricular outflow tract obstruction, such as acute mitral regurgitation or apical thrombus (12). Moreover, performing an echocardiogram at the time of the inciting event would also be useful for comparison of myocardial recovery after 6 to 12 weeks.

Single-photon emission computed tomography with thallium-201 chloride and iodine-123 betamethyl iodophenyl pentadecanoic acid performed on day 4 in this case confirmed the presence of viable myocardium. This is to be expected based on the classic appearance of TC and the small territory involved in SCAD. Because the mechanism of TC is stunning, there is an expectation that the involved myocardium will recover (13), often within weeks to months. Single-photon emission computed tomography (particularly thallium) contributes to radiation exposure and would likely not change management in this case of SCAD, where there was not an indication for revascularization in the absence of ongoing ischemia, hemodynamic instability, or left main dissection (9). Cardiac magnetic resonance may more clearly provide a delineation between TC and ischemia related to SCAD (14), but again, this is unlikely to change management in this case.

Finally, with a confirmed diagnosis of SCAD, it is highly plausible that an underlying diagnosis of fibromuscular dysplasia (FMD) may be contributing to pathology in this presentation. Full-body cross-sectional imaging with computed tomography angiography (a more sensitive modality for FMD than magnetic resonance angiography) is recommended to assess for involvement of other vascular beds at risk (15) but is still unlikely to change clinical management in the acute setting. Although medical therapy would likely remain similar, the diagnosis of FMD would argue for screening for brain aneurysms, which would potentially alter clinical outcomes (16). Enrolling patients in an established registry for SCAD can be of use for improving the future management of the disease (17).

In conclusion, this case underscores the important role of different invasive and noninvasive diagnostic modalities to establish complex diagnoses. It also highlights that multiple processes may occur simultaneously. In turn, developing a relevant differential diagnosis and making a conscious effort to avoid anchoring on a diagnosis is of the essence. Further understanding of the mechanisms of TC, SCAD, and myocardial bridge is pertinent to avoid misdiagnosis of these syndromes, and clinicians should have a heightened awareness that, because of overlapping mechanisms, they may present together.

## Funding Support And Author Disclosures

The authors have reported that they have no relationships relevant to the contents of this paper to disclose.
